# Supplementary material for: DIP/Dpr interactions and the evolutionary design of specificity in protein families
Source: Nat Commun. 2020 May 1;11:2125. doi: 10.1038/s41467-020-15981-8 (PMC7195491; doi:10.1038/s41467-020-15981-8)
Supplement: Supplementary file 1 — Supplementary Information [file 41467_2020_15981_MOESM1_ESM.pdf]

Supplementary Information

DIP/Dpr interactions and the evolutionary design of specificity in protein families

Sergeeva et al.

Protein	Dpr4	Dpr6	Dpr10	Dpr11	DIP-ɣ	DIP-α
Uniprot ID	Q59DX6	M9PC40	M9PF17	Q8MRE6	Q9VAR6	Q9W4R3
1	D74	N102	N82	N146	K67	G70
2	R75	K103	K83	K147	N68	Y71
3	A76	T104	T84	S148	K69	R72
4	S78	S106	A86	S150	G71	G74
5	I80	I108	I88	I152	L73	L76
6	R81	R109	R89	R153	R74	K77
7	K82	H110	H90	L154	A75	A78
8	R83	R111	R91	R155	S76	D79
9	D84	D112	D92	D156	D77	T80
10	L85	I113	L93	G157	Q78	K81
11	H86	H114	H94	H158	T79	A82
12	I87	I115	I95	I159	V80	I83
13	V90	V118	V98	V162	L83	I85
14	G91	G119	G99	D163	Q84	H86
15	L93	Y121	Y101	A165	R86	N89
16	Y95	Y123	Y103	F167	V88	I91
17	T96	T124	T104	I168	T89	T92
18	N97	S125	T105	A169	H90	H93
19	D98	D126	D106	D170	N91	N94
20	Q99	Q127	Q107	Q171	A92	P95
21	R100	R128	R108	R172	R93	R96
22	Q120	Q148	Q128	Q191	R112	S115
23	R122	R150	R130	R193	S114	E117
24	D123	D151	D131	D194	D116	D118
25	E128	E156	E136	E199	M120	M123
26	Q130	Q158	Q138	Q201	Q122	Q125
27	S132	S160	S140	S203	N124	N127
28	T133	T161	T141	T204	T125	T128
29	E134	Q162	Q142	E205	S126	D129
30	P135	P163	P143	P206	P127	P130
31	K136	V164	V144	K207	M128	M131
32	S138	S166	S146	S209	K130	S133
33	G140	F168	S148	R211	V132	I135

Family-wide numbering of interfacial positions

UniProt numbering

Supplementary Table 1: The correspondence between protein specific and family-wide numbering. Family wide-numbering (as defined in Fig. 2A) is compared to numbering of amino acid residues of Dpr4, Dpr6, Dpr10, Dpr11, DIP-γ, and DIP-α in UniProt database.

Binding affinity change

-3.00 ΔΔG, kcal/mol +3.00

stabilizing destabilizing

Interaction	Mutation (a)	Mutation (b)	See footnote	Experiment	FoldX	mCSM	BeAtMusic	Mutabind	Rosetta flex	BindProfX
Dpr6/DIP-α	Dpr6 H110K	Dpr6 H7K	(c)	1.91	0.46	0.91	0.61	1.45	0.83	1.59
	DIP-α K81Q	DIP-α K10Q	(d)	1.31	1.06	0.88	0.89	1.39	-0.03	0.58
	DIP-α S133D	DIP-α S32D	(e)	0.21	0.52	0.59	0.51	0.43	0.17	0.00
	DIP-α G74S	DIP-α G4S	(e)	1.01	0.86	1.41	0.64	0.59	1.21	2.48
	DIP-α A82T	DIP-α A11T	(d)	0.85	1.90	0.54	0.55	0.90	1.06	1.08
	DIP-α N94D	DIP-α N19D	(d)	-0.45	0.12	0.52	0.33	1.03	0.06	0.89
	DIP-α G74A	DIP-α G4A	(d)	-0.46	-0.28	1.12	0.10	0.20	0.80	2.11
	DIP-α G74L	DIP-α G4L	(d)	1.35	6.72	1.47	-0.10	2.29	4.63	2.51
	DIP-α A78K	DIP-α A7K	(d)	-0.95	-0.26	1.01	1.30	0.58	-0.19	1.12
	DIP-α I91A	DIP-α I16A	(d)	2.18	1.79	0.72	1.80	1.77	0.97	0.76
Dpr4/DIP-η	Dpr4 K82H	Dpr4 K7H	(c)	0.12	0.12	0.08	0.19	0.70	-0.34	0.87
DIP-α/DIP-α	DIP-α K81Q	DIP-α K10Q	(d)	-0.12	1.71	3.06	0.92	1.87	-0.06	0.58
	DIP-α G74S	DIP-α G4S	(e)	0.51	0.07	3.04	0.04	1.03	1.76	0.00
	DIP-α S133D	DIP-α S32D	(e)	0.70	0.06	0.84	0.60	0.94	0.46	0.00
Dpr10/DIP-α	Dpr10 Q138D	Dpr10 Q26D	(d)	1.36	3.82	-0.47	1.13	2.51	2.20	0.83
	DIP-α K81Q	DIP-α K10Q	(d)	1.85	1.49	1.01	0.75	1.05	0.78	0.58
	DIP-α D129S	DIP-α D29S	(e)	0.16	-0.85	1.46	1.13	0.61	-0.24	1.61
	DIP-α G74S	DIP-α G4S	(e)	0.65	-1.07	1.26	0.38	0.41	1.01	2.48
	DIP-α S133D	DIP-α S32D	(e)	0.32	0.26	0.50	0.47	0.36	0.20	0.00
	DIP-α I91A	DIP-α I16A	(d)	2.11	1.27	0.84	1.97	1.44	0.87	0.76
	DIP-α G74L	DIP-α G4L	(d)	1.88	6.55	1.27	-0.29	1.92	3.36	2.51
	DIP-α A82T	DIP-α A11T	(d)	1.33	0.87	0.30	0.36	0.76	0.33	1.08
	DIP-α N94D	DIP-α N19D	(d)	-0.13	0.11	0.21	0.24	0.79	0.34	0.89
	DIP-α G74A	DIP-α G4A	(d)	-0.42	-0.25	0.96	-0.12	0.17	0.80	2.11
	DIP-α A78K	DIP-α A7K	(d)	-1.09	-0.47	1.19	1.17	0.48	-0.09	1.12
				PCC	0.54	-0.13	0.19	0.61	0.49	0.05
				RMSE	1.38	1.21	1.56	0.92	1.41	1.40

(a) Protein specific residue numbering of all the mutants as in Uniprot database (see Supplementary Table 1 for details)

(b) Family-wide residue numbering used for interfacial positions throughout the paper (as indicated in Fig. 2)

(c) Value calculated based on previously published SPR binding affinity (K_D) measurements for wild-type (WT) and single mutant (MT) proteins using the following formula: $\Delta\Delta G = RT \ln (K_{D(MT)} / K_{D(WT)})$, Cosmanescu et al. Neuron (2018).

(d) Value calculated based on SPR binding affinity (K_D) measurements for wild-type (WT) and single mutant (MT) proteins using the following formula: $\Delta\Delta G = RT \ln (K_{D(MT)} / K_{D(WT)})$. The supporting SPR data can be found in Supplementary Fig. 1.

(e) Value for the indicated mutation (X) was calculated in the context of a background mutation (DIP-α K81Q). Mutation X is >12Å away from the background mutation (K81Q), allowing us to compare effect of the computed single X mutation to the experimental value, which is calculated based on SPR binding affinity (K_D) measurements for mutant DIP-α K81Q (context) and double mutant DIP-α K81Q X (X) proteins using the following formula: $\Delta\Delta G = RT \ln (K_{D(X)} / K_{D(context)})$.

Pearson correlation coefficient (PCC) and lower root mean square error (RMSE) are calculated for every method (FoldX, mCSM, BeAtMusic, Mutabind, Rosetta flex, BindProfX) based on comparison of the theoretically calculated 25 datapoints with the set of 25 experimental values (Experiment).

Supplementary Table 2: Performance of computational tools to predict changes in binding affinity for a set of DIP and Dpr mutants. Higher Pearson correlation coefficient (PCC) and lower root mean square error (RMSE) reflect better agreement with experimental values (see methods). ΔΔG values are color-coded as indicated in the scale accompanying the data. Calculations were performed on the following PDB structures: 5EO9, 6EG0, 6EFY, 6NRQ (chains C,D).

A

Protein	Mutation	K_D (SPR) to a cognate **DIP-α** partner, μM	ΔΔG (SPR), kcal mol^{-1}	ΔΔG (FoldX), kcal mol^{-1}
**Dpr6**	wild-type	2.3	0	0
**Dpr6**	H7K {H110K}	57.5	1.91	0.46 ± 0.20
**Dpr6**	H7K V31K {H110K V164K}	>300	>2.89	1.24 ± 0.64

B

Protein	Mutation	K_D (SPR) to non-cognate **DIP-α** partner, μM
**Dpr4**	wild-type	>1000
**Dpr4**	K7H {K82H}	>400
**Dpr4**	K7H K31V {K82H K136V}	44.9

C

Protein	Mutation	K_D (SPR) to cognate **DIP-η** partner, μM	ΔΔG (SPR), kcal mol^{-1}	ΔΔG (FoldX), kcal mol^{-1}
**Dpr4**	wild-type	83.7	0	0
**Dpr4**	K7H {K82H}	50.9	-0.29	-0.05 ± 0.03
**Dpr4**	K7H K31V {K82H K136V}	>400	0.93	2.02 ± 0.09

D

Protein	Mutation	K_D (SPR) to non-cognate **DIP-η** partner, μM
**Dpr6**	wild-type	>1000
**Dpr6**	H7K {H110K}	>1000
**Dpr6**	H7K V31K {H110K V164K}	>700

Supplementary Table 3: Comparison of experimental SPR measurements with FoldX data validating predictions of negative constraints in blue and green DIP/Dpr subfamilies. Effect of mutations on **(A)** DIP-α/Dpr6 binding in SPR; **(B)** DIP-α/Dpr4 binding in SPR; **(C)** DIP-η/Dpr4 binding in SPR; **(D)** DIP-η/Dpr6 binding in SPR. DIPs and Dprs are color-coded according to the subgroups they are in Figure 1B. Family-wide residue numbering is followed by UniProt numbering (in curly brackets) for each mutant. FoldX calculations were performed on cognate complexes.

	Dpr11,15,16,17	Dpr6,10	Dpr8,9,21	Dpr13,14,18,19,20	Dpr12	Dpr1,2,3,4,5,7	Dpr7
DIP-γ		Dpr: 7, 14, 16, 29, 31<br>DIP: 9, 10, 16	Dpr: 7, 12, 14, 16, 29, 31<br>DIP: 9, 11, 15	Dpr: 14, 16, 29, 31<br>DIP: 7, 9, 10, 11, 18, 29	Dpr: 13, 14, 16, 29, 31<br>DIP: 7, 9, 11	Dpr: 14, 16<br>DIP: 7, 18	Dpr: 13, 14, 16, 27<br>DIP: 5, 7, 16, 29, 33
DIP-α	Dpr: 10, 29, 31<br>DIP: 6, 9, 11, 15, 22		Dpr: 12, 29, 31<br>DIP: 4, 5	Dpr: 15, 31<br>DIP: 4, 5, 7, 9, 10, 18, 29	Dpr: 7, 10, 13, 18, 20, 31<br>DIP: 4, 7, 9, 10, 11, 29	Dpr: 7, 29, 31<br>DIP: 4, 18, 23	Dpr: 2, 7, 13, 27, 29, 31<br>DIP: 3, 4, 6, 7
DIP-β,λ	Dpr: 12, 29, 31<br>DIP: 6, 9, 11, 15	Dpr: 12, 29, 31<br>DIP: 5		Dpr: 29<br>DIP: 5, 7, 9, 10, 29	Dpr: 10, 12, 13, 31<br>DIP: 7, 9, 10, 11, 29	Dpr: 12, 29, 31<br>DIP: 5, 7	Dpr: 12, 13, 27, 29, 31<br>DIP: 5, 6, 7
DIP-ε,ζ	Dpr: -<br>DIP: 6, 9, 10, 11, 15, 31	Dpr: 31<br>DIP: 10, 13, 30, 31	Dpr: 29<br>DIP: 5, 10, 13, 31		Dpr: 13, 31<br>DIP: 5, 7, 9, 11, 31, 32, 33	Dpr: 33<br>DIP: 10, 13, 18, 31	Dpr: 13, 33<br>DIP: 5, 6, 10, 13, 18, 31, 32, 33
DIP-δ	Dpr: 10, 14, 16, 17, 29, 31<br>DIP: 6, 15, 19, 22	Dpr: 7, 10, 18, 20, 31<br>DIP: 5, 10	Dpr: 7, 10, 12, 20, 31<br>DIP: 19	Dpr: 29, 31<br>DIP: 5, 7, 10, 29		Dpr: 7, 10, 29, 31<br>DIP: 5, 9	Dpr: 7, 10, 29, 31, 32<br>DIP: 5, 6, 18, 33
DIP-ι,θ,η	Dpr: 10<br>DIP: 9, 15	Dpr: 31<br>DIP: 10	Dpr: 1, 12, 29, 31<br>DIP: 5, 11, 20	Dpr: 31<br>DIP: 5, 7, 9, 10, 11, 29	Dpr: 10, 13, 31<br>DIP: 9, 11, 23, 29		Dpr: -<br>DIP: 5, 18
DIP-κ	Dpr: 2, 10, 27, 32<br>DIP: 5, 9, 15, 22	Dpr: 2, 27, 29, 31, 32<br>DIP: 10	Dpr: 12, 27, 29, 31, 32<br>DIP: 5, 11, 15	Dpr: 29, 31<br>DIP: 5, 7, 9, 10, 11, 29	Dpr: 10, 27, 31, 32<br>DIP: 5, 9, 11, 23, 29	Dpr: 32<br>DIP: 5, 22	

Supplementary Table 4: Negative constraints predicted between non-cognate DIP/Dpr subfamilies. Interfacial positions destabilizing non-cognate binding are listed on both Dpr and DIP side in off-diagonal elements. Interfacial positions correspond to family-wide numbering presented in Fig.2 and compared to Uniprot numbering in Supplementary Table 1.

Mutant	Forward primer sequence	Reverse primer sequence
Dpr10 V144K	gaatagctgcgcttaggctgcgtggatatttggcact	agtgccaaatatccacgcagcctaagcgcagctattc
Dpr10 V144K Q142E	gtgccaaatatccacggagcctaagcgcagctattccgtc	gacggaatagctgcgcttaggctccgtggatatttggcac
Dpr10 V144K Q142E L93G	cacggtgaggatgtgcccgtcacgatgtcgtatc	gatacgacatcgtgacgggcacatcctcaccgtg
Dpr11 K207V	cacgggcgctgactacgggctccgtagaga	tctctacggagcccgtagtcagcgcccgtg
Dpr11 K207V E205Q	acgggcgctgactacgggctgcgtagagacctgg	ccaggtctctacgcagcccgtagtcagcgcccgt
Dpr11 K207V E205Q G157L	cggtcaagatgtgtagatctcgcagacgaatccaggacac	gtgtcctggattcgtctgcgagatctacacatcttgaccg
DIP-ɣ D77T	gagctaaaacggtctgagtggaggctctcagccaac	gttggctgagagcctccactcagaccgttttagctc
DIP-ɣ R86N	gcattatgggtgacaacgttaccttggagagctaaaacggt	accgttttagctctccaaggtaacgttgtcacccataatgc
DIP-ɣ R112S	ctaaaaatcagcaaactgagcgaaagtgaccgcggctgc	gcagccgcggtcactttcgctcagtttgctgatttttag
DIP-α K81Q	tcaaggccgacacccaggccattcaagcc	ggcttgaatggcctgggtgtcggccttga
DIP-α A82T	aaggccgacaccaagaccattcaagccatcc	ggatggcttgaatggtcttggtgtcggcctt
DIP-α K81Q S133D	gaacacggatccgatgaaggatcagattggcttcctggac	gtccaggaagccaatctgatccttcatcggatccgtgttc
DIP-α K81Q G74S	cggctatcgggtgagctggctcaaggc	gccttgagccagctcacccgatagccg
DIP-α I91A	tcaagccatccacgagaacgtagccacgcacaatcctc	gaggattgtgcgtggctacgttctcgtggatggcttga
DIP-α N94D	aacgtaatcacgcacgatcctcgcgtcacgg	ccgtgacgcgaggatcgtgcgtgattacgtt
DIP-α G74A	gctatcgggtggcctggctcaaggc	gccttgagccaggccacccgatagc
DIP-α G74L	ggcggctatcgggtgctatggctcaaggccgac	gtcggccttgagccatagcacccgatagccgcc
DIP-α A78K	ggtgggctggctcaagaaggacaccaaggccattc	gaatggccttggtgtccttcttgagccagcccacc
DIP-α K81Q D129S	caatctgactcttcatcggactcgtgttcagttggcacatat	atatgtgccaactgaacacgagtccgatgaagagtcagattg

Supplementary Table 5: Forward and reverse primer sequences used for the mutants in the study. All positions are numbered as in Uniprot. Uniprot IDs for the proteins are given in Supplementary Table 1.

A

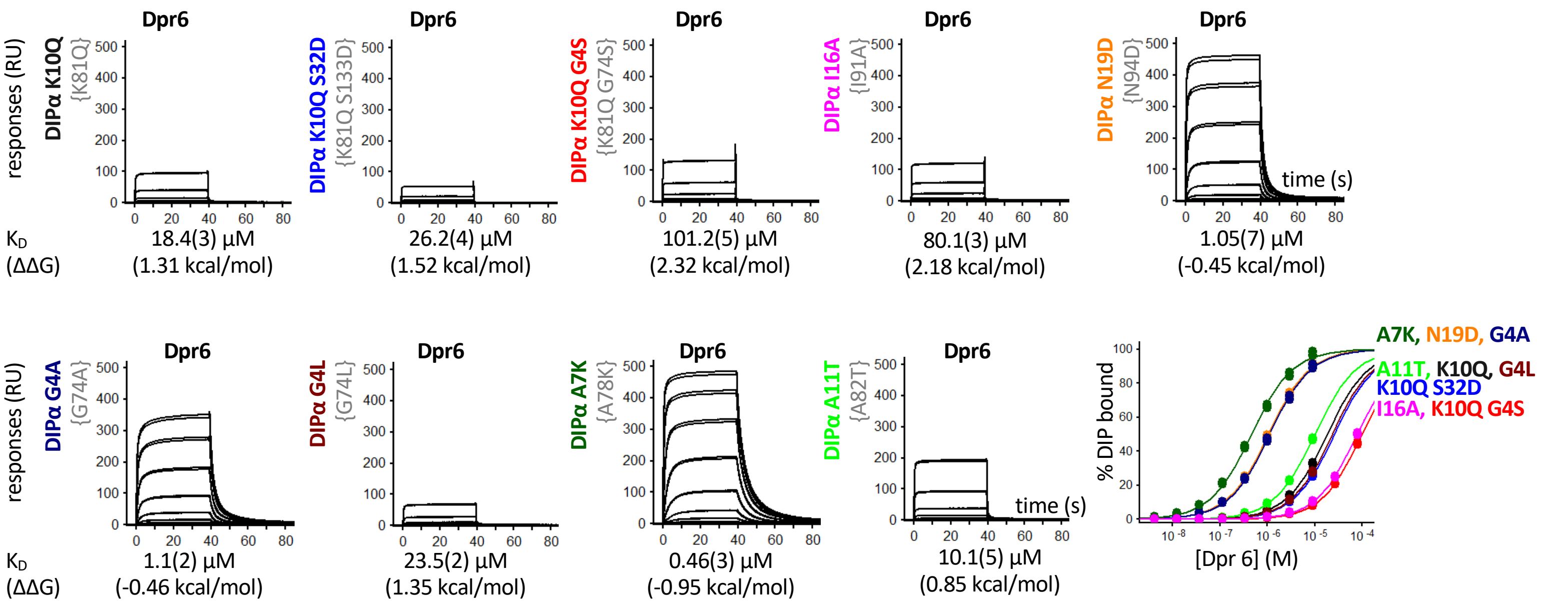

B

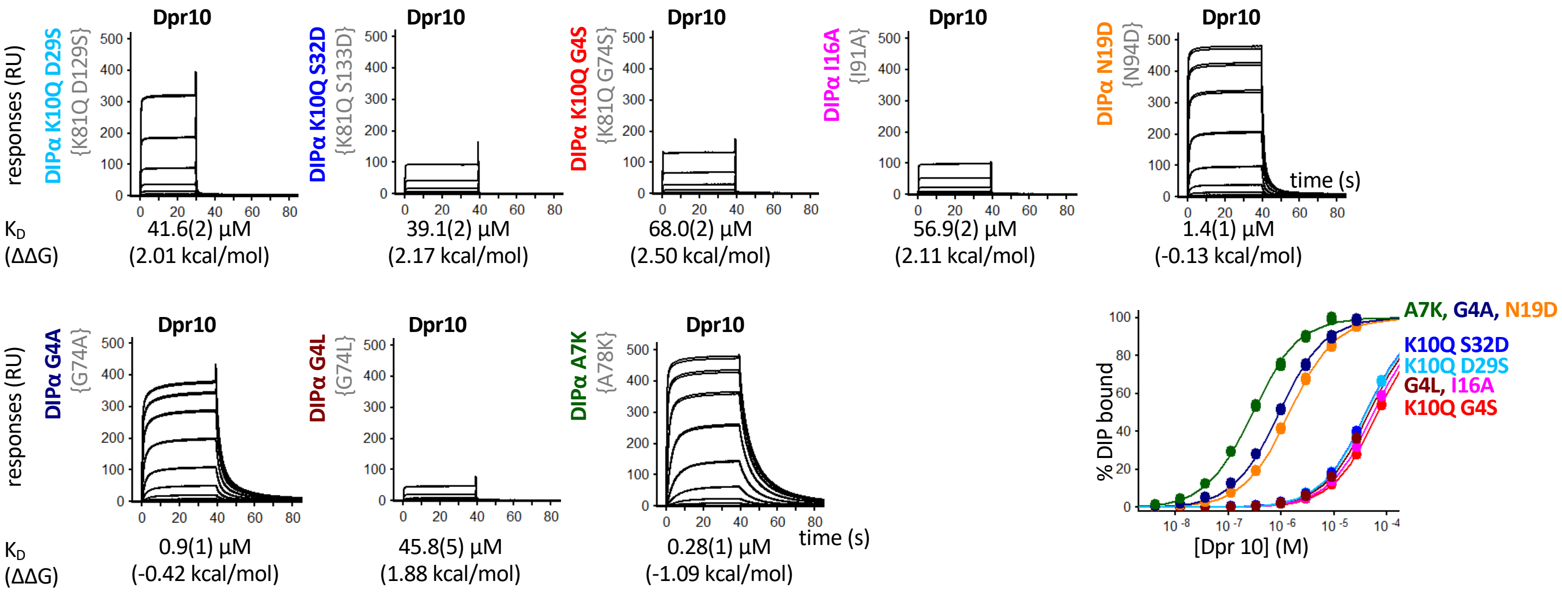

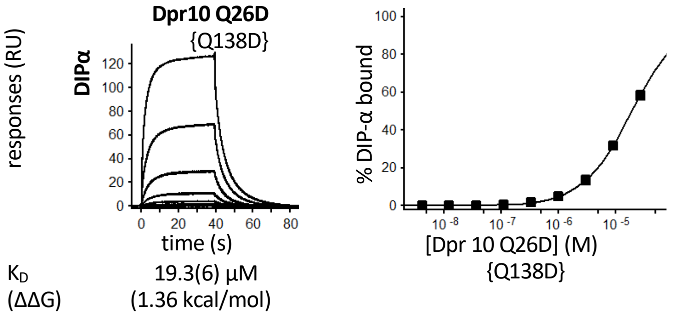

C

Protein	Oligomeric state	K_D dimerization (μM)
DIP-α K10Q {K81Q}	dimer	19.7 ± 1.9
DIP-α K10Q G4S {K81Q G74S}	dimer	46.3 ± 5.7
DIP-α K10Q S32D {K81Q S133D}	dimer	64.4 ± 3.9

Supplementary Fig. 1: SPR and AUC experiments of mutants presented in Supplementary Table 2. Effect of mutations on **(A)** DIP-α/Dpr6 binding in SPR; **(B)** DIP-α/Dpr10 binding in SPR; and **(C)** DIP-α/DIP-α binding in AUC. Analyte Dpr proteins were flown over the chip surface with immobilized DIPs (A and B). K_D values of DIP/Dpr interactions are given below the SPR sensorgrams. The number in parenthesis represents the fitting error in the last significant figure, in μM, for a single experiment with an expected experimental error up to 15%. Binding isotherms are shown to the right of sensorgrams. Family-wide residue numbering is followed by UniProt numbering (in curly brackets) for each mutant. AUC data presented as the mean of two independent measurements, with errors ± the difference of each of these from the mean. Source data are provided as Source Data file

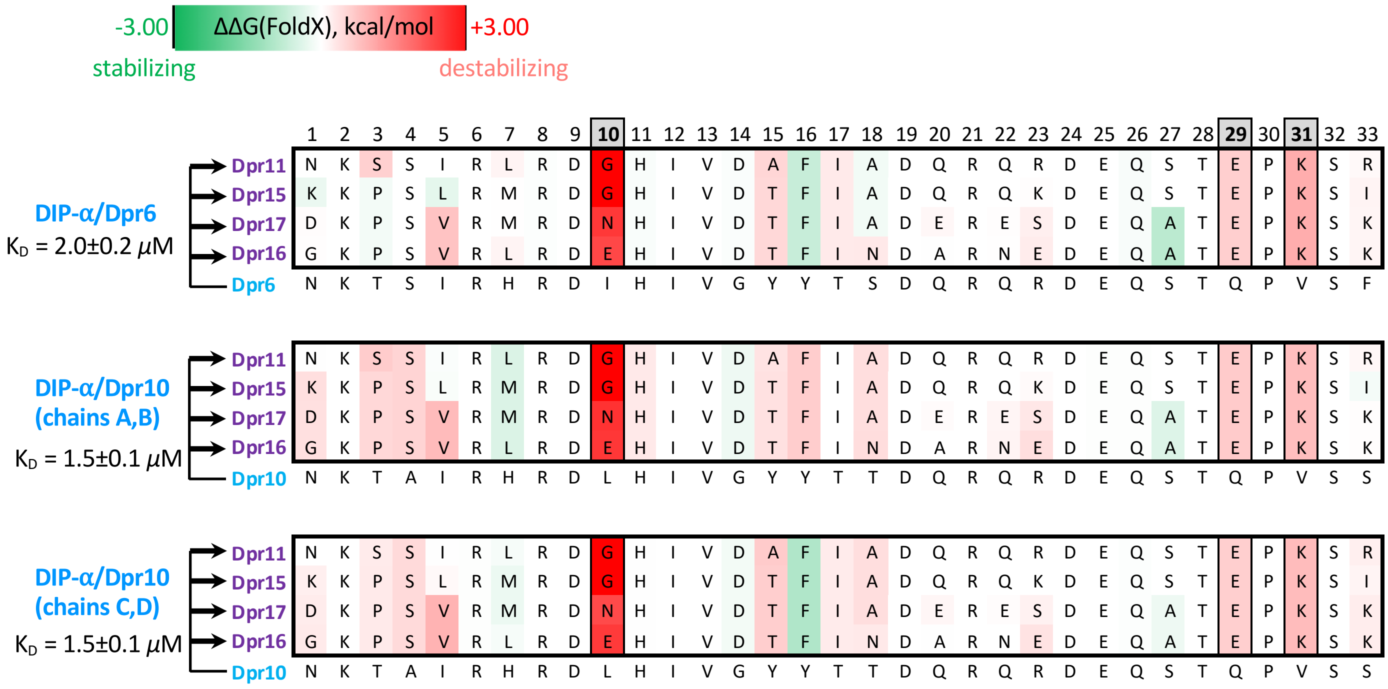

Supplementary Fig. 2: Extended version of Fig. 3A. Negative constraints on the purple Dpr surface, which prevent inter-subgroup binding of the four purple group Dprs with DIP-α are assigned based on consensus of predictions using three representative blue group complexes: DIP-α/Dpr6, and the two distinct conformations observed in crystals of DIP-α/Dpr10. Positions that pass both energy and evolutionary filters are shown in grey. All notations as in Fig. 3A. Average K_D values ± standard deviation are given for each interaction based on a number of independent SPR experiments, see methods. Source data are provided as Source Data file.

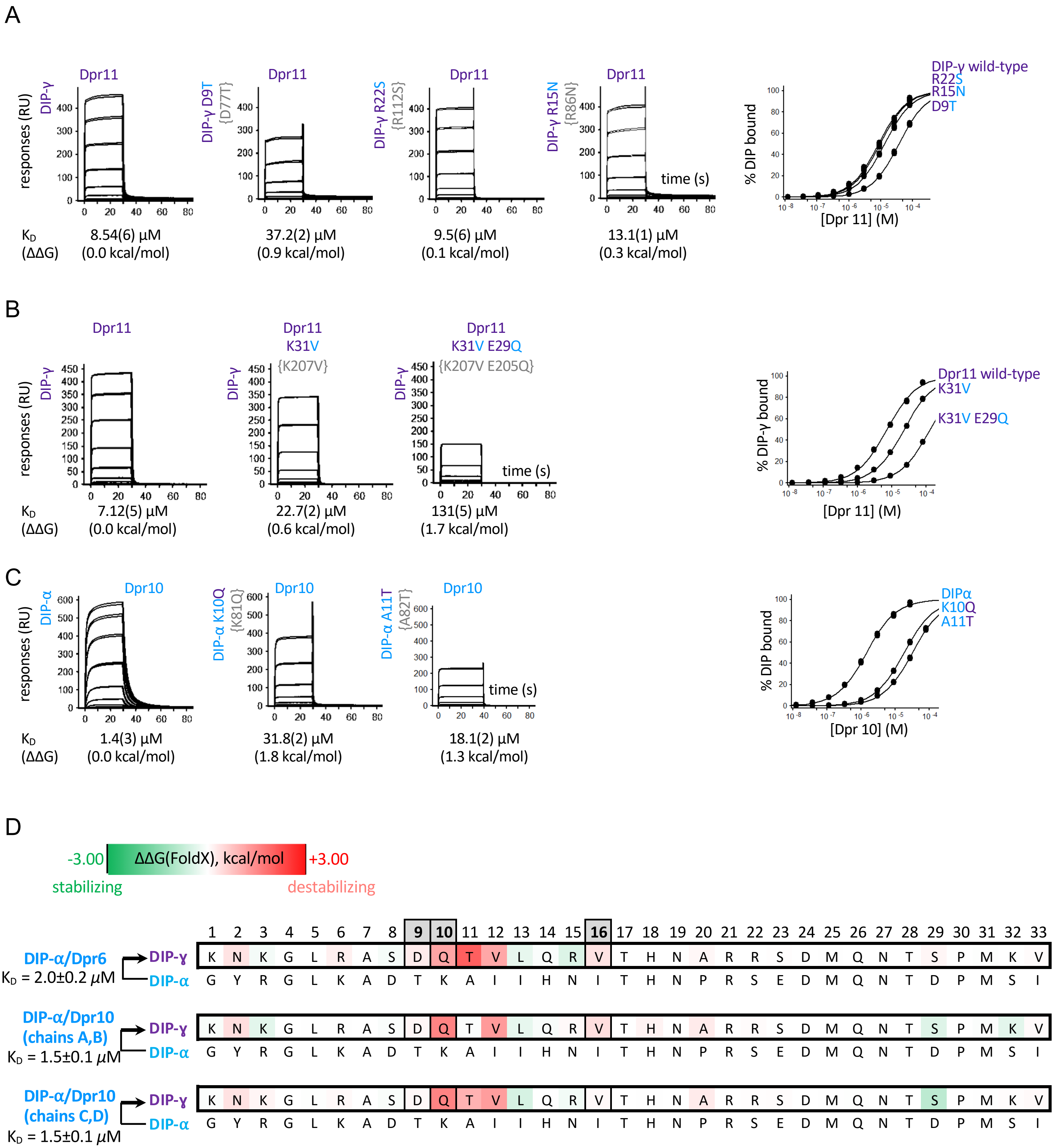

Supplementary Fig. 3: SPR experiments supporting prediction of negative constraints that prevent blue to purple inter-subgroup binding on (A) DIP-α surface, (B) blue Dpr surface, (C), DIP-γ surface. All notations as in Fig. 3C. Each row shows SPR sensorgrams of Dpr analytes binding over individual DIP-immobilized surfaces. An overlay of the binding isotherms for each surface is shown to the right. K_D (ΔΔG) values for each DIP/Dpr interaction are listed below the SPR sensorgrams. For each K_D, the number in parenthesis represents the fitting error in the last significant figure, in μM, for a single experiment with an expected experimental error up to 15%. Family-wide numbering of interfacial positions is used for all the mutants. Uniprot numbering is given in curly brackets. **(D).** Extended version of Fig. 4C. Negative constraints on the DIP-γ surface, which prevent inter-subgroup binding to the blue group Dprs are assigned based on consensus of predictions using three representative blue group complexes: DIP-α/Dpr6, and the two distinct conformations observed in crystals of DIP-α/Dpr10. All notations as in Fig. 3A. Average K_D values ± standard deviation are given for each interaction based on a number of independent SPR experiments, see methods. Source data are provided as Source Data file

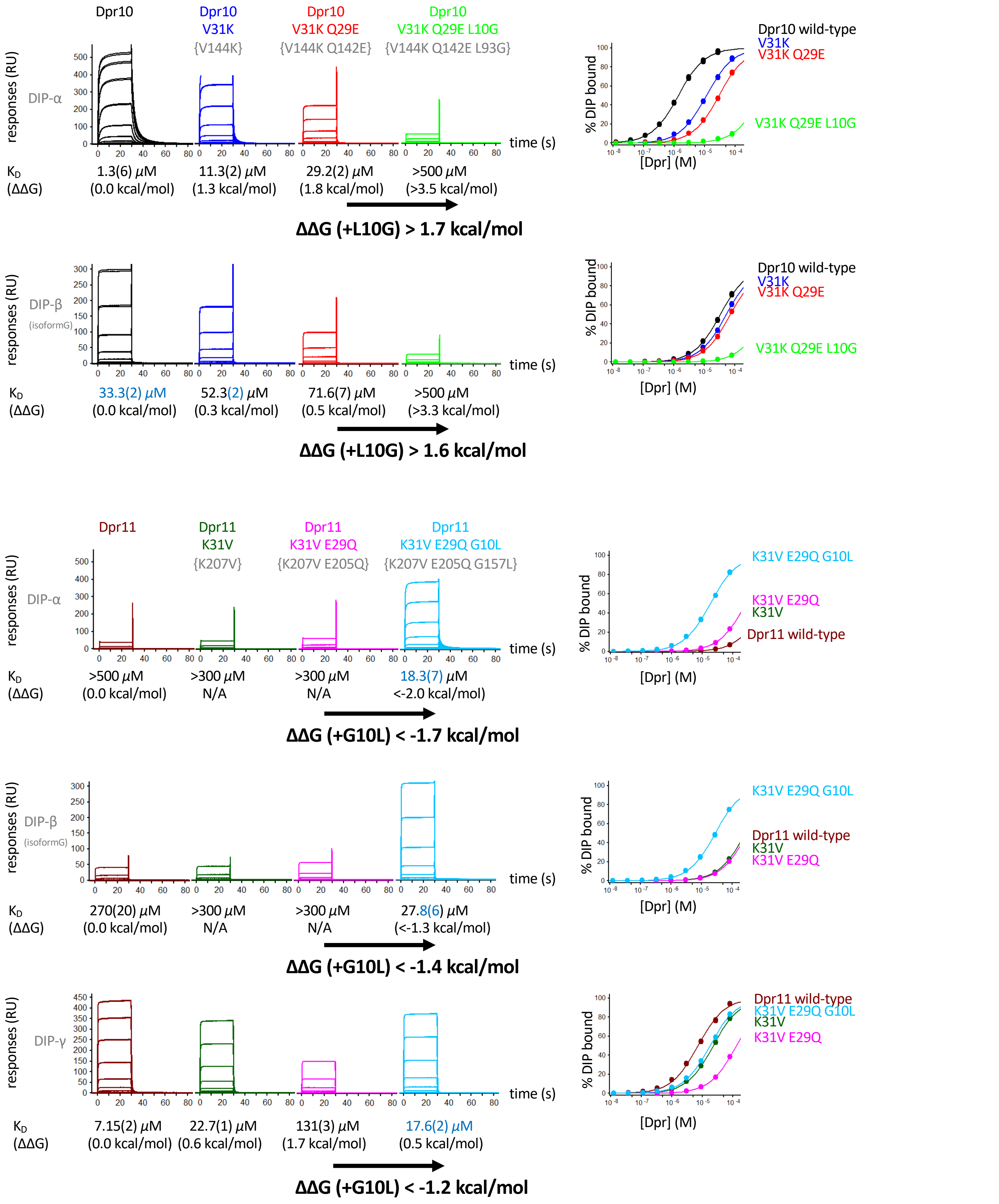

Supplementary Fig. 4: SPR experiments of the binding of Dpr10, Dpr11, and their mutants to DIP-α, DIP-β, and DIP-γ. Each row shows SPR sensorgrams of Dpr analytes binding over an individual DIP-immobilized surface. Binding isotherms of DIP/Dpr interactions are given to the right of SPR sensorgrams. K_D values are given under each sensorgram. The number in parenthesis represents the fitting error in the last significant figure, in μM, for a single experiment with an expected experimental error up to 15%. Family-wide numbering of interfacial positions is used for all the mutants. Uniprot numbering is given in curly brackets. Source data are provided as Source Data file
