# Supplementary material for: DIP/Dpr interactions and the evolutionary design of specificity in protein families
Source: Nat Commun. 2020 May 1;11:2125. doi: 10.1038/s41467-020-15981-8 (PMC7195491; doi:10.1038/s41467-020-15981-8)
Supplement: Supplementary file 3 — Description of Additional Supplementary Information [file 41467_2020_15981_MOESM3_ESM.pdf]

Description of Additional Supplementary Files

File Name: Supplementary Data 1
Description: FoldX effects for 3044 mutations of 33 interfacial residues of 13 template complexes on the DIP and Dpr side into residues occurring in DIPs and Dprs of other subfamilies. All $\Delta\Delta G$ binding energy differences are in kcal mol 1. Negative constraints for 42 combinations of non-interacting DIP and Dpr subgroups that passed both energy and evolutionary filters are highlighted in grey. All other notations as in Fig. 3A.
