# Supplementary material for: DIP/Dpr interactions and the evolutionary design of specificity in protein families
Source: Nat Commun. 2020 May 1;11:2125. doi: 10.1038/s41467-020-15981-8 (PMC7195491; doi:10.1038/s41467-020-15981-8)
Supplement: Supplementary file 5 — Reporting summary [file 41467_2020_15981_MOESM5_ESM.pdf]

nature research

Corresponding author(s): Barry Honig, Lawrence Shapiro

Last updated by author(s): March 18, 2020

Reporting Summary

Nature Research wishes to improve the reproducibility of the work that we publish. This form provides structure for consistency and transparency in reporting. For further information on Nature Research policies, see Authors & Referees and the Editorial Policy Checklist .

Statistics

For all statistical analyses, confirm that the following items are present in the figure legend, table legend, main text, or Methods section.

n/a	Confirmed	
☐	☒	The exact sample size (*n*) for each experimental group/condition, given as a discrete number and unit of measurement
☐	☒	A statement on whether measurements were taken from distinct samples or whether the same sample was measured repeatedly
☐	☒	The statistical test(s) used AND whether they are one- or two-sided<br>*Only common tests should be described solely by name; describe more complex techniques in the Methods section.*
☒	☐	A description of all covariates tested
☒	☐	A description of any assumptions or corrections, such as tests of normality and adjustment for multiple comparisons
☒	☐	A full description of the statistical parameters including central tendency (e.g. means) or other basic estimates (e.g. regression coefficient) AND variation (e.g. standard deviation) or associated estimates of uncertainty (e.g. confidence intervals)
☒	☐	For null hypothesis testing, the test statistic (e.g. *F*, *t*, *r*) with confidence intervals, effect sizes, degrees of freedom and *P* value noted<br>*Give P values as exact values whenever suitable.*
☒	☐	For Bayesian analysis, information on the choice of priors and Markov chain Monte Carlo settings
☒	☐	For hierarchical and complex designs, identification of the appropriate level for tests and full reporting of outcomes
☐	☒	Estimates of effect sizes (e.g. Cohen's *d*, Pearson's *r*), indicating how they were calculated

Our web collection on statistics for biologists contains articles on many of the points above.

Software and code

Policy information about availability of computer code

Data collection	No software was used for data collection of DIP/Dpr protein sequences from Uniprot (2019) and NCBI (2019) databses. Similarly, DIP/Dpr protein structures were also retrieved manually from RCSB Protein Data Bank (2019)
Data analysis	Calculating effects of mutations on protein-protein binding: FoldX (version 4c), mCSM (2014), BeAtMusic (version 1.0), Mutabind (2016), Rosetta flex ddG (2018), BindProfX (2017)<br>Sequence-based predictions of specificity residues: GroupSim(2008), SDPpred (2004), SPEER (2012), Multi-harmony (2010)<br>Structure alignment and visualization: Pymol (version 2.2.0)<br>Homology models: MODELLER (v9.18) and scwrl4.0 (2009)<br>Sequence logo preparation: WebLogo (version 3.6.0)<br>Ortholog search: reciprocal best hit BLAST (version 2.2.28)<br>Protein sequence filtering and processing: CD-HIT (version 4.7), Jalview (version 2.0), Clustal-Omega (2011)<br>SPR data processed using Scrubber 2.0 (BioLogic Software)<br>AUC data processed using SednTerp (version 1.10, Alliance Protein Laboratories) and HeteroAnalysis software (version 1.1.60) packages<br>Pearson correlation coefficient and Root mean square errors were computed in Excel

For manuscripts utilizing custom algorithms or software that are central to the research but not yet described in published literature, software must be made available to editors/reviewers. We strongly encourage code deposition in a community repository (e.g. GitHub). See the Nature Research guidelines for submitting code & software for further information.

Data

Policy information about availability of data

All manuscripts must include a data availability statement. This statement should provide the following information, where applicable:

- Accession codes, unique identifiers, or web links for publicly available datasets
- A list of figures that have associated raw data
- A description of any restrictions on data availability

The raw data underlying Fig 3 and Supplementary Figs 1-4 are provided as a Source Data file. PDB accession codes for structures used in this study: 5EO9, 6NRQ, 6NRR, 6EG0, 6NRW, 6EG1. All other relevant data is available from the corresponding authors upon reasonable request.

Field-specific reporting

Please select the one below that is the best fit for your research. If you are not sure, read the appropriate sections before making your selection.

- [x] Life sciences
- [ ] Behavioural & social sciences
- [ ] Ecological, evolutionary & environmental sciences

For a reference copy of the document with all sections, see nature.com/documents/nr-reporting-summary-flat.pdf

Life sciences study design

All studies must disclose on these points even when the disclosure is negative.

Sample size	We include all 21 Dprs proteins and 11 DIP proteins of Drosophila Melanogaster in our study, use all of the available DIP/Dpr crystal structures (available in PDB), and all sequences of DIP/Dpr orthologs available in NCBI dataset for Insecta species, as of September 2019.
Data exclusions	We established our criteria for exclusion as follows. The sequences of DIPs and Dprs were manually curated to get rid of redundancy arising from multiple GI (GenInfo Identifier) numbers pointing to the same protein product. The obtained non-redundant set of full-length sequences was further processed to remove sequences that had non-complete Ig1 domains (as this domain contained the needed info on interfacial positions required for the analysis.
Replication	All FoldX calculations are averaged over 10 independent calculations for every mutation. The AUC experiments are representative of two technical replicates. For several wild type interactions discussed in this manuscript the SPR values represent averages based on 5-6 replicate experiments with experimental error ranging from 10 to 15%. A similar level of experimental error (10-15%) is expected for the KDs of all other Dpr or DIP mutants determined in this study, where each concentration was tested in duplicate but in a single experiment. All attempts of replication were successful.
Randomization	Grouping of DIP/Dpr into binding subgroups was based primarily on the strongest heterophilic binding preferences but also on DIP/Dpr sequence similarity
Blinding	Blinding is not relevant to our study. We were not blinded to group allocation during data collection. We used all available crystal structures to date. Assignment of DIP and Dpr sequences in Insecta to specific ortholog groups was performed using unbiased reciprocal best hit BLAST approach.

Reporting for specific materials, systems and methods

We require information from authors about some types of materials, experimental systems and methods used in many studies. Here, indicate whether each material, system or method listed is relevant to your study. If you are not sure if a list item applies to your research, read the appropriate section before selecting a response.

Materials & experimental systems

n/a	Involved in the study	
x		Antibodies
	x	Eukaryotic cell lines
x		Palaeontology
x		Animals and other organisms
x		Human research participants
x		Clinical data

Methods

n/a	Involved in the study	
x		ChIP-seq
x		Flow cytometry
x		MRI-based neuroimaging

Eukaryotic cell lines

Policy information about cell lines

Cell line source(s)	Human: FreeStyle 293-F cells (Thermo Fisher Scientific)
Authentication	Human: FreeStyle 293-F cells used in this study were authenticated by morphology and PCR
Mycoplasma contamination	Human: FreeStyle 293-F cells used in this study were routinely tested and confirmed to be Mycoplasma negative.
Commonly misidentified lines (See ICLAC register)	no commonly misidentified cell lines were used in this study
